# Diversity in United States dementia prevention trials: An updated systematic review of eligibility criteria and recruitment strategies

**DOI:** 10.1002/alz70860_106484

**Published:** 2025-12-23

**Authors:** Najoua Lazaar

**Affiliations:** ^1^ Erasmus Medical Center, Rotterdam, Netherlands

## Abstract

**Background:**

Given the elevated dementia risk in underrepresented demographic groups in the US—particularly in Latino and Non‐Latino Black individuals compared to Non‐Latino White individuals—it is vital that these groups are well‐represented in dementia prevention research. Eligibility criteria and recruitment strategies may play a key role in promoting participant diversity. The aim of this review was to examine eligibility criteria and recruitment strategies in US dementia prevention trials in light of participant diversity.

**Method:**

A systematic review was conducted using Medline (including PubMed), Embase, Cochrane Library and CINAHL. We explored the percent White participants for trials using vs. not using a specific eligibility criterion or recruitment strategy using Hodges‐Lehmann median difference estimation.

**Result:**

Of forty‐four studies meeting the inclusion criteria, twenty‐seven reported on racial/ethnic diversity. Analyses demonstrated that criteria regarding cardiovascular disease, pulmonary disease, hearing impairment, and sedentary lifestyle were associated with relatively high participant diversity, while gastro‐intestinal/liver disease, motivation to participate, and language proficiency criteria were associated with relatively little diversity. The pooled percentage in the updated review was 75,4% for White participants, 4,9% for Black participants, 0,3% for Asian participants, 0,00% for American Indian/American Native participants, 0,00% for Native Hawaiian and Pacific Islander participants and 3,8% for participants with another or unspecified background. A pooled percentage of 4,1% Latino participants was included. These proportions are not representative of the US population. Information on recruitment strategies was often lacking. Three studies described recruitment efforts explicitly aimed at increasing diversity. Recruitment strategies associated with relatively high racial/ethnic diversity included recruitment via referral/word‐of‐mouth, television/radio advertising, and recruitment at church.

**Conclusion:**

Eligibility criteria could be improved by revisiting and revising how they are defined (e.g. researcher's judgement about motivation to participate). Regarding recruitment, several recommendations are provided, including 1) lifting barriers to study participation (e.g. through reimbursement), 2) collaborating with community partners, and 3) formally studying the effectiveness of recruitment strategies.

